# Future Training Pathways in Percutaneous Coronary Interventions: Interventional Critical Care, Complex Coronary Interventions, and Interventional Heart Failure

**DOI:** 10.1016/j.jacadv.2024.101338

**Published:** 2024-10-14

**Authors:** Saraschandra Vallabhajosyula, Mirvat Alasnag, Konstantinos Dean Boudoulas, Laura J. Davidson, Robert T. Pyo, Robert F. Riley, Pinak B. Shah, Poonam Velagapudi, Wayne B. Batchelor, Alexander G. Truesdell, Critical Care Cardiology Working Group of the American College of Cardiology Interventional Section Leadership Council

**Affiliations:** a Section of Cardiology, Department of Medicine, Warren Alpert Medical School of Brown University, Providence, Rhode Island, USA; b Brown University Health Cardiovascular Institute, Providence, Rhode Island, USA; c Cardiac Center, King Fahd Armed Forces Hospital, Jeddah, Saudi Arabia; d Division of Cardiovascular Medicine, Department of Medicine, Ohio State University Wexner Medical Center, Columbus, Ohio, USA; e Bluhm Cardiovascular Institute, Northwestern University Feinberg School of Medicine, Chicago, Illinois, USA; f Division of Cardiology, Department of Medicine, Stony Brook University Renaissance School of Medicine, Stony Brook, New York, USA; g Overlake Medical Center and Clinics, Bellevue, Washington, USA; h Division of Cardiovascular Medicine, Department of Medicine, Brigham and Women’s Hospital, Harvard Medical School, Boston, Massachusetts, USA; i Division of Cardiovascular Medicine, Department of Medicine, University of Nebraska School of Medicine, Omaha, Nebraska, USA; j Inova Schar Heart and Vascular, Inova Fairfax Medical Campus, Falls Church, Virginia, USA; k Virginia Heart, Falls Church, Virginia, USA

**Keywords:** chronic total occlusion, critical care cardiology, heart failure, interventional cardiology, percutaneous coronary intervention

## Abstract

While there has been a proliferation of training and practice paradigms in the realm of noncoronary interventions, coronary disease remains the predominant pathology necessitating interventional cardiology expertise. The landscape of coronary disease has also experienced a significant transformation due to rapidly evolving technologies, clinical application of mechanical circulatory support and other device innovations, and increasing acuity and complexity of patients. The modern interventional cardiologist is subject to challenges including decreasing coronary procedural volume, need to maintain clinical and financial productivity, and often also requirements of continued scholastic pursuit. Therefore, in the coming decade, there will be greater impetus to develop expertise in multiple new domains of practice. In this document, we propose 3 training paradigms that may assist the tertiary/quaternary center coronary interventional cardiologist to develop a unique clinical/scholastic niche, maintain clinical skills and productivity, and develop care models for complex patients within local and regional tertiary/quaternary hospitals.

## Highlights

• Coronary disease has higher complexity, rapidly evolving procedural and imaging technologies, and increasing acuity and complexity of patients.
• This review discusses 3 training paradigms in interventional critical care cardiology, complex and high-risk coronary interventions, and interventional heart failure.
• These pathways may help develop a unique clinical/scholastic niche, maintain clinical skills and productivity, and develop care models for complex patients.

Percutaneous coronary intervention (PCI) was first performed in 1977.^1^ Since the first procedure where balloon angioplasty was used to dilate a coronary stenosis in a stable patient, the percutaneous treatment of coronary artery disease (CAD) has evolved significantly and rapidly over the past 5 decades.^2^ In parallel with advances in technology, PCI has been adopted for increasingly complex clinical and anatomical subsets such as reduced ventricular function and heart failure (HF), chronic total occlusions (CTO), heavily calcified lesions, patients ineligible for surgical revascularization, cardiogenic shock, and cardiac arrest.^3^, ^4^, ^5^, ^6^ Increased risk profiles of patients treated with PCI stem not only from cardiovascular complexity but also from increased burden of important noncardiovascular comorbidities. Many more patients now have advanced age and frailty, as well as diabetes, and significant kidney, liver, neurologic, and peripheral arterial diseases that raise the risks of major procedural complications, to include in-hospital mortality.^7^^,^^8^ In this manuscript, we seek to highlight the changes to the practice of coronary interventions, highlight financial and procedural volume considerations that impact “hub” centers, and discuss 3 unique training pathways for the future practice of coronary interventions.

## Current and future landscape

### Percutaneous coronary intervention

Presently, there are many patients who require high-risk PCI following a multidisciplinary heart team approach to decision-making and management.^9^ It has been demonstrated that patients are less likely to be offered PCI when they are considered high surgical risk, even though individual patients in this cohort may experience significant benefits from revascularization.^10^^,^^11^ There are several reasons why patients in this higher risk group may not be offered PCI—to include concerns regarding the ability to adequately hemodynamically support the patient during complex intervention without cardiopulmonary bypass, lack of familiarity with contemporary calcium modification devices and techniques, and lack of data surrounding these complex high-risk patients who are often largely excluded from clinical trials.^6^ The use of mechanical circulatory support (MCS) has permitted many more complex patients to undergo PCI safely and effectively.^12^ Patients who are survivors of cardiac arrest can present a complex clinical conundrum, often necessitating immediate decision-making, where some may benefit from emergent extracorporeal life support whereas others may possess multiple unfavorable features that portend a poor long-term prognosis where it may be most prudent to defer high-risk revascularization.^13^^,^^14^ Currently, the pathway for trainees to become an interventional cardiologist involves a mandatory 1-year fellowship program that, while variable among training programs, involves training in PCI, as well as some exposure to structural, congenital, and peripheral interventions. MCS may be utilized to differing degrees by different programs depending on patient complexity and local practice patterns. However, with the advent of a greater need for complex PCI, greater use of MCS, and a growing HF and complex comorbid patient population, there is interest in further subspecialization within interventional cardiology (IC).^15^

### Changes in practice of medicine in health care systems

In parallel with these changes in clinical practice, the definitions of academic and clinical medicine continue to evolve. Historically, academic medicine was limited to large university hospitals, with federally funded translational researchers. However, there has been increasing consolidation of health care systems that involve both academic and private hospitals to regionalize care and share resources to better serve patients and communities.^16^^,^^17^ This has resulted in the development of “central hubs,” often in a “hub-and-spoke” model, to provide tertiary and quaternary services to complex patients while ensuring judicious use of human and financial resources.^3^^,^^18^^,^^19^ In the contemporary era, fewer than 50% of cardiology leaders seek the classical physician-scientist to staff hub hospitals.^20^ Unlike previously salaried models that ensured adequate protected time for basic-science or clinical research and medical education, the modern physician at the “hub” hospital is often required to be involved more clinically on a day-to-day basis.^21^ Many physicians at larger centers, both academic and large private practices, are required to demonstrate clinical productivity, meet clinical relative value units targets, and contribute to the clinical mission of health care systems affiliated to medical schools.^21^ Work relative value units are often used to determine salary and bonus structures across the spectrum of clinical medicine, both academic and private. In the era of lower PCI numbers, and longer duration of individual cardiac procedures due to progressively increasing case complexity, it is challenging to continue to practice solely as an interventional cardiologist. In smaller private practices, many physicians supplement their IC role with significant general cardiology responsibilities, that is, clinic visits, hospital consults, interpreting echocardiogram and nuclear studies, and performing and interpreting peripheral vascular studies. However, within large cardiovascular medicine practices, divisions/departments often have clearer distinction of roles for noninvasive versus invasive physicians.

Additionally, these referral hubs have other unique considerations. They are often superspecialized and serves as a regional referral sites for very complex acute and elective patients.^3^^,^^18^ However, the financial margins at many tertiary/quaternary centers have been threatened over recent years due to decreased reimbursement in parallel with increasing complexity of patient care among increasingly comorbid patients.^22^ The need to maintain subspecialty and focused expertise in IC also must be balanced against the need to improve access to patient care.^23^ Therefore, divisional leaders at academic and large private practice centers will continue to face the strain from these financial forces and will be required to redesign traditional paradigms. Some creative strategies include fixed salaried structures, development of nonacademic health care systems to offset academic medicine, mergers and acquisitions, and development of academic relative value units and/or “time-value units” (which account for nonrevenue generating but valuable time- and labor-intensive clinical and nonclinical work) have been proposed.^24^^,^^25^ However, the bulwark of clinician reimbursement will continue to rely on clinical productivity for the foreseeable future.

## Novel training pathways

**Figure 1:**
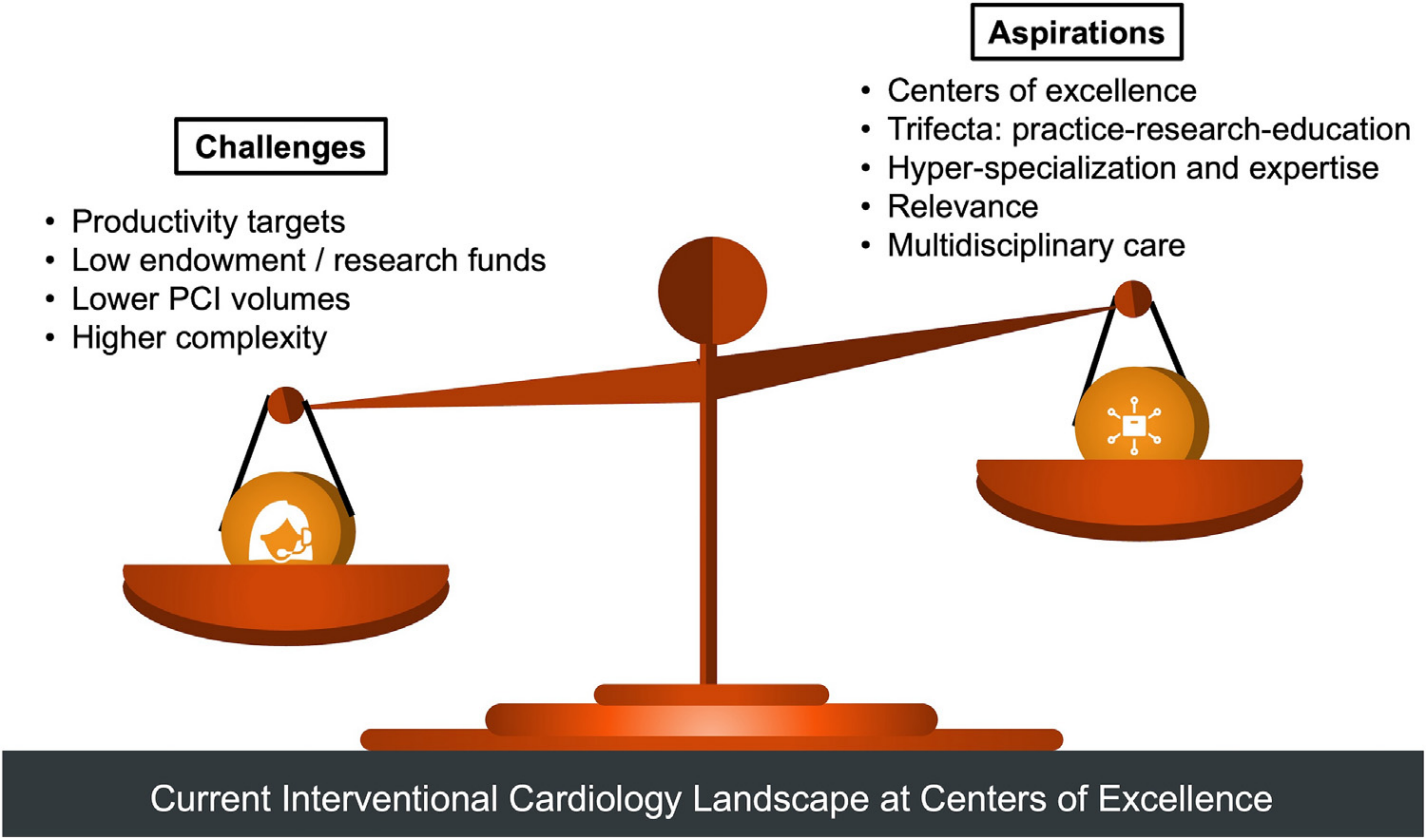
Current Interventional Cardiology Landscape at Tertiary/Quatenary Medical Centers PCI = percutaneous coronary intervention.

Considering the above, IC practice may need to be reimagined to remain relevant everywhere (Figure 1). Current training programs for structural, peripheral, and complex coronary interventions frequently involve an additional year of training as noted in the recent American College of Cardiology (ACC) Advanced Training Statement for Interventional Cardiology.^26^ However, for the interventionalist who prefers to perform coronary interventions and develop a unique and increasingly relevant niche, the following alternative training paradigms may be relevant. While these are not mutually independent, they focus on different aspects of IC that all complement coronary interventions and the management of patients with CAD (Central Illustration). The Critical Care Cardiology Working Group of the ACC Interventional Section Leadership Council (ACC ISLC) sought to elaborate on these novel training pathways. This was proposed by the lead author (S.V.) and vetted by the Critical Care Cardiology Working Group of the ACC ISLC, the larger ACC ISLC, and endorsed by the ACC ISLC. Input from other ACC councils or non-ACC organizations was not sought. This viewpoint has been authored by members of the ACC ISLC to summarize their individual and collective experiences to provide guidance for trainees who are seeking to distinguish themselves along these pathways.

### Interventional and critical care cardiology

Compared to historical controls, the contemporary cardiac intensive care unit (CICU) population has more frequent requirement for noncardiac organ support.^7^^,^^27^ Emerging evidence has demonstrated that the presence of high-intensity staffing models involving clinicians with critical care expertise in the CICU is associated with improved outcomes.^28^ As a consequence, there has been enthusiasm for the field of critical care cardiology and dual training in cardiovascular medicine and critical care medicine.^29^, ^30^, ^31^ Despite the clearly established clinical need for such physicians, there remains a paucity of dedicated critical care cardiologists.^32^^,^^33^ In 2023, per the American Board of Internal Medicine, while there were over 25,000 certified cardiologists and 14,000 critical care medicine physicians, there were fewer than 160 physicians board certified in both specialties.^34^

Despite the growing presence of critical care cardiology, most CICUs in this country continue to be staffed by interventional cardiologists and/or noncardiac intensivists.^35^ The combination of interventional critical care cardiology has instinctive appeal since the cardiac catheterization laboratory (CCL) is often the first point-of-contact for acute cardiovascular emergencies.^33^ Frequently, in the CCL and the CICU, physicians are required to opine on vascular access, hemodynamic assessment, insertion of indwelling pulmonary artery catheters, hemodynamic support (pharmacological and mechanical) strategies, procedural sedation, and palliative and end-of-life care.^32^^,^^36^ Procedural performance in the CICU is challenging due to patient acuity, inadequate ergonomics, and spatial limitations. The interventionalist has the unique advantage of higher volume of procedures and vascular access at the inception of their career and troubleshooting difficult anatomies and complications—which offers clear benefits that translate to procedural performance in the CICU.^32^

From a staffing standpoint, interventional cardiologists are ubiquitously available at most large medical centers, specifically those housing a level I CICU.^34^^,^^36^^,^^37^ In the CICU, the interventional cardiologist is also well suited to provide ongoing care for acute coronary syndrome, cardiogenic shock, cardiac arrest, and postprocedure complications, making them a natural fit for the CICU environment. Academically and administratively, this provides the early career interventional cardiologist the unique opportunity to participate in and lead multidisciplinary care teams.^32^^,^^38^ By virtue of their dual training, these physicians may have opportunities to build these care paradigms, serve as system leaders for acute cardiovascular care expansion (to include regional spoke-and-hub networks), and serve as an internal referral resource for other physicians.

Current training pathways for critical care cardiology involve sequential training in both subspecialties, however, recently, there has been increasing discussion of hybrid training pathways of interventional and critical care cardiology.^32^, ^33^, ^34^ Due to the severe supply-demand mismatch, a certification pathway for recognition in critical care cardiology without formal board certification has also been proposed.^34^ These pathways may provide a meaningful training alternative in the modern era to address this clinical gap and expand services across the country. An alternate interventional and critical care cardiology pathway with 4 years of combined postgraduate training after internal medicine has also been proposed.^33^^,^^39^ This pathway, while suitable to achieve IC board certification, may not meet the time requirements set forth by the American Board of Internal Medicine for critical care medicine board certification.

### Complex high-risk and chronic total occlusion PCI

While the terms “complex CAD” or “high-risk CAD” have not been formally defined, they encompass both complex anatomic lesions and clinical parameters including advanced age, frailty, comorbidities, significant lesion calcification, compromised hemodynamic status, depressed ventricular function, and concomitant valvular disease.^40^, ^41^, ^42^ Such features increase both the procedural complexity of PCI and the risk of adverse patient outcomes.^5^ This has led to novel techniques, devices, and data surrounding “complex high-risk and indicated PCI (CHIP)” procedures to meet this need.^4^ Complex coronary interventions frequently require close collaboration with colleagues within cardiothoracic surgery, cardiac imaging, and a deeper understanding of cross-sectional imaging to understand coronary and vascular anatomy, vascular access, PCI strategies, collateral circulation, and to characterize CTO anatomy.

Despite their high prevalence, these lesions have historically comprised a small subset of PCI procedures, due to both ambiguity in indications, increased procedural risk, and lack of specialized training and procedural tools.^40^^,^^41^ However, over the past decade, there have been significant strides in crossing algorithms, equipment, and dedicated training directed toward CTO PCI that have led to improved procedural success and lower procedural adverse event rates.^43^, ^44^, ^45^ Given the direct correlation between operator volume and outcomes, formalized training in CTO PCI (and adjunctive coronary computed tomography angiography for case planning and performance) and higher annual procedural volumes can help operators care for this population when clinically indicated.^46^ Importantly, the use of CTO PCI toolbox has significant advantages in solving complex coronary situations in non-CTO cases.^47^

The direct correlation between increased volumes and improved outcomes is also seen with non-CTO lesions, such as, severe calcification, left main PCI, and MCS-supported PCI.^48^, ^49^, ^50^ Due to the increased risk of these procedures, higher-risk operators must be knowledgeable, confident, and rehearsed in managing diverse complications ranging from coronary perforations to hemodynamic collapse, lest uncertainty regarding complication management contribute to inadvertent and unwanted undertreatment of patients with high-complexity coronary disease. This often involves purposeful practice and ongoing individual and team mental and physical rehearsal regarding training with complication management devices (covered stents, coils, thrombin, MCS, etc) and algorithms for troubleshooting common and uncommon problems.^51^^,^^52^ Lastly, in addition to standard intracoronary imaging and physiology, advanced techniques such as near-infrared spectroscopy and microvascular physiology may fall within the purview of the complex coronary operator.^53^^,^^54^

Despite the increasing prevalence of complex CAD, there has not been commensurate evolution of formal training efforts aimed at treating this expanding patient population.^55^ Presently, there are only a few formal programs in the United States dedicated to advanced CHIP training, with no formal application process. Formal training requirements for these types of fellowships have been proposed, although no consensus has been reached.^56^ For graduates that complete these programs, studies have demonstrated their ability to perform complex procedures in complex patients on a routine basis with close interdisciplinary collaboration with the larger heart team.^46^^,^^57^

### Interventional heart failure

The rapid increase in pharmacologic, percutaneous, and surgical therapies in HF coupled with the temporal increase in the prevalence of HF has led to the interest and evolution of interventional HF (IHF) as a new subspecialty.^32^^,^^58^ IHF focuses on the evaluation and management of patients with complex and advanced HF utilizing advanced clinical skills and the ability to perform diagnostic and therapeutic procedures.^58^^,^^59^ These physicians serve as hybrid specialists capable of bridging the gap between procedural skills in the CCL and clinical skills for the treatment of complex and advanced HF.

IHF specialists are familiar with invasive hemodynamics including the utilization of right heart catheterization adjuncts such as vasoactive medications or physiologic exercise challenge to make a detailed diagnosis and tailored plan for therapy. They can place pulmonary artery pressure sensors and manage the patient with HF based on interpretation of the data and clinical evaluation.^60^ IHF specialists have the knowledge to determine when a patient may require cardiac transplantation or durable left ventricular assist device and how to best select and manage these patients.^32^^,^^58^ In patients with cardiogenic shock, the IHF specialist has the clinical expertise and understanding of cardiovascular physiology to determine when and what type of acute percutaneous MCS device is needed, and the ability to perform these procedures.^58^^,^^59^ With the increasing adoption of percutaneous MCS in the CCL, the IHF specialist is well placed to aid in device selection algorithms including insertion of venoarterial extracorporeal membrane oxygenation.^61^^,^^62^ Furthermore, these specialists are experts at understanding peripheral limb perfusion catheters, left ventricular unloading, and other unique considerations to MCS that require integration of complex hemodynamics and a mix of IC and HF expertise.^63^^,^^64^

With the development of transcatheter devices for the treatment of valvular heart disease, particularly in patients with HF, the IHF specialist is well suited to diagnose and manage these patients as well. For example, the IHF specialist may be particularly well positioned to understand the timing of transcatheter interventions including transcatheter edge-to-edge repair and transcatheter aortic valve replacement in advanced HF patients.^59^^,^^65^ Patients with systolic and/or diastolic HF can have increase in left atrial and pulmonary pressures resulting in debilitating symptoms and exercise intolerance. Devices have been developed to create a permanent interatrial shunt decompressing the left atrium.^65^ The recent RELIEVE-HF (REducing Lung congestIon symptoms using the V-wavE shunt in AdVancEd Heart Failure) trial, presented at the ACC Scientific Sessions (and yet to be published), noted a neutral signal for outcomes from interatrial shunt placement with variability based on baseline ventricular function.^66^ Furthermore, new percutaneously delivered device therapies for HF are constantly emerging and include left ventricular reconstruction, ventriculoplasty, annuloplasty, superior vena cava occlusion to reduce cardiac filling pressures, autonomic modulation (eg, baroreflex activation therapy, vagus nerve stimulation, splanchnic nerve modulation), respiratory modulation (eg, phrenic nerve stimulation), and myocardial gene therapies, among others.^60^ IHF specialists also possess specialized knowledge and skills to provide longitudinal HF care for patients with and without durable MCS or cardiac transplantation and lead HF programs or quality improvement initiatives within their cardiovascular groups or institutions.

## Current landscape, limitations, and future directions

While these pathways offer unique opportunities for clinical distinction, academic advancement, and leadership roles, the current data on the practice patterns of such physicians are still evolving. In a survey of 120 dual-trained cardiology and critical care physicians, a majority obtained certification through the practice pathway.^35^ Nearly 30% of the physicians who obtained certification through the practice pathway or fellowship were trained in IC.^35^ Over 50% of these respondents felt they regularly used their critical care skills in the care of CICU patients including airway and ventilator management, end-organ failure, and renal replacement therapy.^35^ In recent years, there has been increasing emphasis to align training pathways with practice requirements stratified by tier of CICU care to decrease redundancy of training and increase the CICU workforce.^34^^,^^67^ In a survey of 545 CTO operators and 190 IC fellows with an interest in CHIP/CTO PCI (41% from the United States), median case volumes were 205 (IQR: 150-328) and 20 (IQR: 5-50) for total PCIs and CTO PCIs, respectively.^68^ Of these physicians, 34% worked at academic institutes, 31% trained CHIP fellows, and 34% had dedicated CTO PCI days in clinical practice.^68^ In a recent survey of 54 IHF physicians (78% from the United States), 100%, 54%, and 29% trained for 7, 8, and >8 postgraduate years, respectively.^69^ Of these, 61% were in academic practice, 83% trained in HF prior to IC, 82% felt they would repeat this unique training pathway, and 86% would recommend it to current trainees.^69^ Chief areas of primary focus were CAD (42%) and cardiogenic shock (42%), with 74% physicians spending ≤0.5 full-time equivalents in the care of advanced HF and cardiac transplantation patients.^69^ Currently, graduates of such pathways are eligible to obtain board certification for critical care medicine and HF in addition to IC boards. However, CHIP/CTO training (like structural heart disease and peripheral vascular disease) remains nonaccredited. It is likely that these board certifications may be influenced by the new cardiovascular medicine board that is being proposed.^70^

**Figure 2:**
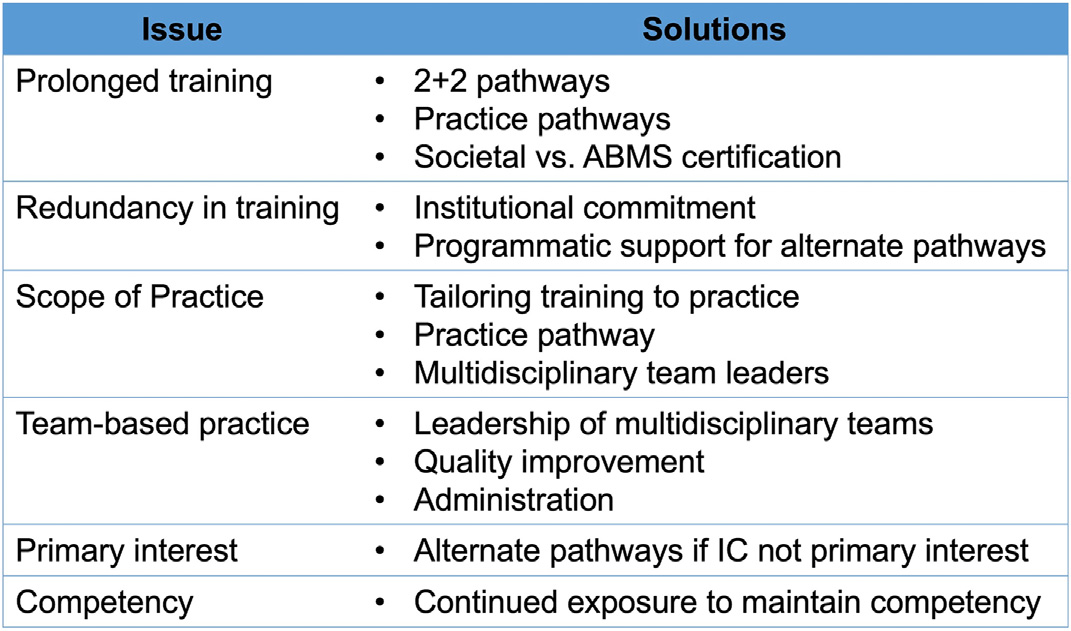
Limitations of Proposed Future Paradigms for Academic Interventional Cardiology ABMS = American Board of Medical Specialties; IC = interventional cardiology.

Despite the ongoing interest, several limitations currently exist to the adoption of these training models (Figure 2). These physicians typically train for longer periods than their contemporaries and therefore face associated challenges to include prolonged debt burden, long working hours, and training in aspects of the individual subspecialties that might not be fully relevant to their final scope of practice.^32^ They often have to participate individually in both the IC and subspecialty (critical care cardiology, HF, CHIP/CTO PCI) call pools in parallel, which may result in greater cumulative weeknight and weekend call resulting in higher rates of burnout and physical and mental exhaustion. Given the challenges of maintaining appropriate clinical volumes, these physicians are required to maintain ongoing competency in multiple procedural and cognitive competencies, which requires life-long learning and participation in multiple different and individually demanding institutional and national quality programs.^71^

**Central Illustration:**
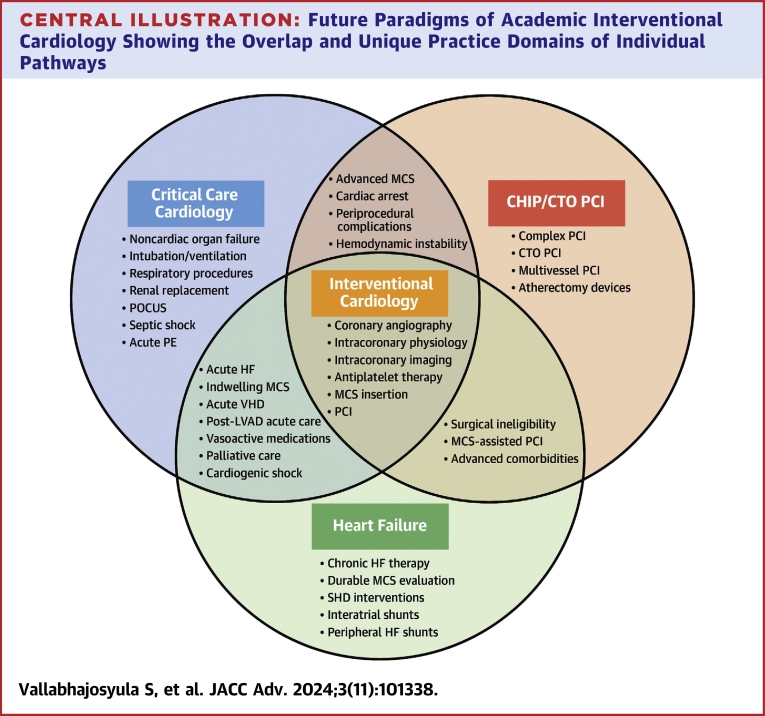
Future Paradigms of Academic Interventional Cardiology Showing the Overlap and Unique Practice Domains of Individual Pathways CTO = chronic total occlusion; HF = heart failure; LVAD = left ventricular assist device; MCS = mechanical circulatory support; POCUS = point-of-care ultrasonography; PE = pulmonary embolism; SHD = structural heart disease; VHD = valvular heart disease; other abbreviation as in Figure 1.

These futuristic pathways might benefit from significant programmatic redesign with an abbreviated foundational training pathway.^72^^,^^73^ During clinical practice, continued education in the form of proctorships can supplement the training in order to ensure graduates are competent in individual cognitive and procedural skills and tasks. The role, value, and contribution of such specialists to the contemporary team-based management models need further systematic evaluation. Furthermore, the differences in the resources, local practice patterns, presence of a hub-and-spoke model, and the absence of local mentorship, might make it challenging for some graduates to thrive in different health care systems.^34^ Future directions for training must incorporate novel educational strategies such as simulation education, virtual grand rounds, hands-on “on-the-job” training, proctoring programs, and continued medical education courses.^74^ However, the availability of these opportunities is heterogenous, and a need exists for structured training (initial and lifelong) formats for both trainees and postgraduate physicians.

## Conclusions

PCI has undergone significant evolution over the past few decades, with an increasing focus on addressing complex subsets. However, the current landscape of PCI faces challenges, including an aging population, rising comorbidities, and diminished reimbursement. Large hub hospitals—private, academic, and hybrid—play a pivotal role in delivering high-quality care to the most critically ill patients. While interventional cardiologists at these centers have responsibilities to include research and education, most of the reimbursement still derives from clinical productivity, limiting their ability to take on additional roles. Recognizing that interventional cardiologists with dual training may be uniquely positioned to meet the needs of such patients, there is a pressing need to explore innovative training pathways, such as interventional critical care cardiology, IHF, and complex coronary interventions. These approaches may mitigate burnout from prolonged training and offer early career interventional cardiologists a unique opportunity to establish themselves as local and national leaders, actively engaging and leading multidisciplinary care teams, and establishing innovative care paradigms.

## Funding support and author disclosures

Dr Boudoulas serves as a coinvestigator on NIH Grant R01H157453 and collaborator on NIH Grant R01H167511. Dr Velagapudi serves on the Speakers Bureau for Medtronic and Shockwave; and on the advisory board for Medtronic. Dr Pyo serves as a speaker for Abiomed and Boston Scientific; and as an advisor for Medtronic. Dr Davidson is a site principal investigator and receives research funding from Edwards Lifesciences for CLASP TR trial. Dr Riley serves on the Speakers Bureau for Abbott Vascular, Boston Scientific, and Shockwave Medical; and reports equity in Vantis and Egg Medical. Dr Truesdell serves as a consultant for Abiomed Inc; and on the Speakers Bureau for Abiomed Inc and Shockwave Medical. All other authors have reported that they have no relationships relevant to the contents of this paper to disclose.
